# Clinical Characteristics and Risk Factors for the Recurrence of Benign Paroxysmal Positional Vertigo

**DOI:** 10.3389/fneur.2019.01190

**Published:** 2019-11-13

**Authors:** Cui Ting Zhu, Xing Qquan Zhao, Yi Ju, Yan Wang, Mei Mei Chen, Yu Cui

**Affiliations:** ^1^Department of Neurology, Beijing Tiantan Hospital, Capital Medical University, Beijing, China; ^2^China National Clinical Research Center for Neurological Diseases, Beijing, China; ^3^Center of Stroke, Beijing Institute for Brain Disorders, Beijing, China

**Keywords:** benign paroxysmal positional vertigo, cerebrovascular risk factors, recurrence rate, young, older

## Abstract

Benign paroxysmal positional vertigo (BPPV) manifests itself as a paroxysm of vertigo and nystagmus lasting several seconds, which is self-limiting. The clinical characteristics and risk factors for the recurrence of BPPV in different ages have not yet been investigated.

**Materials and Methods:** A retrospective observational study was conducted in the Department of Neurology in Beijing Tiantan Hospital from July 2009 to June 2015. The study included 1,012 patients aged 18–93 years. All patients received the definitive diagnosis and canalith repositioning maneuvers treatment and finally accomplished follow-up. Demographic variables, potential recurrence risk factors, neurological examination, and laboratory indexes were assessed.

**Data Analyses:** The *t*-test or chi-squared test was first performed for group comparison, then logistic regression analysis was used to investigate the risk factors of BPPV recurrence.

**Results:** The 1-year recurrence rates of BPPV patients after reposition maneuvers were, respectively, 22.79% (aged 18–45 years), 23.92% (aged 45–60 years), 28.89% (over 60 years). The recurrence rates among the three groups have no statistically significant difference. Logistic regression analysis shows that women BPPV patients have more recurrence risks than do men. Ménière's disease (odds ratio = 6.009, 95% confidence interval: 2.489–14.507, *p* < 0.001), hypertension (odds ratio = 1.510, 95% confidence interval: 1.095–2.084, *p* = 0.012), migraine (odds ratio = 2.534, 95% confidence interval: 1.164–5.516, *p* = 0.019), and hyperlipemia (odds ratio = 1.419, 95% confidence interval: 1.024–1.968, *p* = 0.036) were risk factors for the recurrence of BPPV in patients.

**Conclusion:** We conclude that Ménière's disease, hypertension, migraine, and hyperlipemia may be independent risk factors for the recurrence of BPPV in patients, but aging does not increase the recurrence risk.

## Introduction

The objective of this study was to investigate the clinical characteristics and risk factors for the recurrence of benign paroxysml positional vertigo (BPPV) in young, middle-aged, and older patients to provide a guideline for developing effective prevention strategies for BPPV.

BPPV, a common peripheral vestibular disorder, occurs in nearly 17% of patients with dizziness or vertigo (1). It is caused by abnormal stimulation of the cupula in any of the three semicircular canals. Typically, BPPV manifests itself as a paroxysm of vertigo and nystagmus lasting several seconds, which is self-limiting (2). Currently, canalith repositioning maneuvers (CRM) are the mainstream treatment for BPPV, which have been successful in improving symptoms. The recurrence rate of BPPV after treatment is high, which results in anxiety and poor quality of life in patients. Vertigo and dizziness increase the risk of falls because of the imbalance caused by impaired movement and space-directed damage. Therefore, understanding the risk factors for the recurrence of BPPV is imperative for better symptom and relapse prevention.

Previous studies showed that cerebrovascular risk factors influenced BPPV recurrence and severity of symptoms (3). In Taiwan (4), a nationwide population study found that BPPV increased the risk of ischemic stroke. It was also reported that BPPV patients with hypertension and hyperlipidemia were at a higher risk of symptom recurrence (5). One observational study reported that patients with BPPV had a higher prevalence of hypertension and coronary artery disease (4). Another study showed that carotid plaque was a risk factor of the peripheral vestibular disorder (6). It has also been found that hyperglycemia and hyperinsulinemia are risk factors for the recurrence of BPPV (7). Normal aging is accompanied by vascular changes, such as the formation of utriculus that causes hypoperfusion of inner circulation, giving rise to otolith formation. The internal auditory artery and anterior vestibular artery are branches of the anterior inferior cerebellar artery. An ischemic attack of the transitory artery is likely to cause peripheral vestibulopathy (8). However, these risk factors are highly heterogeneous and thus not clearly understood. Therefore, further studies are required to explore how cerebrovascular risk factors influence the recurrence of BPPV.

In addition, studies have shown that symptoms and prognosis differ between young and old BPPV patients. A population study in Germany revealed that almost 1.1 million adults suffered from BPPV each year (9). It was reported that the prevalence of BPPV in the young adults was 9% (10). One study found that older BPPV patients receiving treatment experience residual dizziness, characterized by subjective dizziness and imbalance without episodic nystagmus (11). Residual dizziness may lead to nervousness, panic, or insomnia and negatively affect the quality of life of patients (12). The incidence of psychogenic disorder is influenced by age, being prevalent in individuals over 65 years old. It is well-known that old age is accompanied by a combination of comorbidities. So far, the risk factors for recurrence of BPPV in young patients have not been sufficiently characterized. BPPV is largely a secondary outcome of inner ear disorders such as Ménière's disease, migraine, and sudden deafness (13). Therefore, in this retrospective study, we aimed to (1) investigate the risk factors for BPPV in young, middle-aged, and older patients separately; and (2) explore the association between cerebrovascular risk factors and BPPV recurrence. We hypothesized that the recurrence of BPPV in young, middle-aged, and older BPPV patients may be modulated by different cerebrovascular risk factors.

## Materials and Methods

### Patients and Procedures

The study was performed according to the Declaration of Helsinki guidelines, and written informed consent was obtained from all participants. The patients in our study only underwent standard treatment without additional interventions for research purposes, so no formal ethics approval was required. In total, 1,046 patients were retrospectively enrolled in this study. The patients received the definitive diagnosis and CRM treatment, and finally got cured. Finally, 1,012 patients accomplished follow-up in the Department of Neurology, Beijing Tiantan Hospital from July 2009 to June 2015, with 3.3% lost rate of follow-up (Details in Supplementary Figure 1). After enrollment, the patients were divided into the young group (aged 18–45 years), middle-aged group (aged 45–60 years), and the older group (over 60 years). The patients who met the criteria were enrolled: (1) Vertigo attack due to changes in head position, with a positive Dix-Hallpike or Roll test; (2) Patients were suitable for CRM treatment and finally got cured; (3) Patients who signed the informed consent and accepted a telephone follow-up. Those with the following conditions were excluded: (1) Patients who did not complete position test and CRM; (2) Patient information was incomplete; (3) Patients who did not complete follow-up.

### Treatment and Follow-Up

Following the clinical practice guideline of BPPV established by the American Academy of Otolaryngology-Head and Neck Surgery Foundation (14), the patients were treated with the CRM. The Dix-Hallpike and roll tests were conducted on patients. Patients with abnormal test results were diagnosed with BPPV. Patients with posterior semicircular canal otolith were treated with Epley or Semont maneuver. The Lempert or Barbecue maneuver and the Gufoni method (to the healthy side) were performed for horizontal geotropic nystagmus. The calcium carbonate debris was located in the long arm of the semicircular canal. Horizontal apogeotropic nystagmus: Gufoni maneuver (to the affected side) or modified Semont maneuver was also used. The calcium carbonate debris was attached to the cupulolithiasis. The treatments for multiple BPPV were chosen on the basis of severity of vertigo and nytagmus, and different semicircular canal-type maneuvers were applied. The success of the treatment was defined as the absence of vertigo and nystagmus on positional testing (15). One week after CRM treatment, the BPPV patients returned to the hospital for efficacy evaluation. Recurrence was defined as: After successful treatment for 1 week; (1) The patients had similar positional vertigo as the primary attack; (2) a positive positional nystagmus test was confirmed.

### Measurement Indexes

The patients were assessed in terms of demographic variables, potential recurrent risk factors, neurological examination, and laboratory indexes. A questionnaire was used to record gender, recurrence times (one, two, or more than three), Ménière's disease, sudden deafness, migraine, cerebrovascular risk factors (hypertension, hyperlipemia, diabetes, coronary heart disease, arterial plaque) and cerebral infarction as related data. We identified the etiology of BPPV as idiopathic, various disorders of the inner ear, vestibular neuronitis (patients with a history of acute vestibulopathy on the affected side), prolonged recumbent position, or traumatic. Patients were followed up 1 year after the treatment to record the recurrence data via telephone, which was carried out by a postgraduate clinician. During the assessment, hypertension (ICD-10 code E10.X01), hyperlipemia (ICD-10 code E78.501), diabetes (ICD-10 code E11.951), and coronary heart disease (ICD-10 code I 25.101) were defined according to International Classification of Diseases 10 (ICD-10) (16). Secondary BPPV was defined as the presence of a history of acute or chronic inner ear disease or unilateral vestibular loss within 1 year prior to this study (13). Ménière's disease was defined according to the American Academy of Otolaryngology-Head and Neck Surgery Foundation (1995) guideline (17). Migraine was diagnosed on the basis of the International Headache Society (IHS) criteria (18). Sudden deafness was defined as a history of unilateral sensorineural hearing loss with sudden onset, without other prior otological histories (19).

### Data Analysis

Patients were divided into three different age groups (young patients 18–45 years; middle-aged patients 45–60 years; older patients over 60 years). Statistical significance between the young, middle-aged, and older BPPV patients was determined using a *t*-test, chi-squared test, or Fisher's exact test. When *p* < 0.05, the differences between the three groups were deemed to be statistically significant. Multivariable logistic regression was performed to identify the recurrence risk factors in all of the patients. All statistical analyses were performed using the IBM SPSS statistical software version 22.0.

## Results

### Demographic Profile

In total, 1,012 BPPV patients accepted definitive diagnosis, CRM treatment, and finally accomplished follow-up in the Department of Neurology, Beijing Tiantan Hospital from July 2009 to June 2015. There were 208 young patients (80 male and 128 female), 489 middle-aged patients (146 male and 343 female), and 315 older patients (90 male and 225 female). The average ages of the three groups of patients were 37.10 ± 6.47, 53.63 ± 4.18, and 67.70 ± 5.88 years, respectively (Table 1). The sex ratio difference between the three groups was not statistically significant. The median disease duration of BPPV in the young group was 23.93 ± 46.21 days, in the middle-aged group 40.38 ± 20.78 days, and in the older group 30.90 ± 57.66 days, with no statistically significant difference (*p* = 0.158) (Details in Supplementary Figure 1). We collected the differential type of semicircular canal: posterior canal 155 young patients, 368 middle-aged patients, and 248 older patients; horizontal canal 53 young patients, 121 middle-aged patients, and 67 older patients. Multiple semicircular canal BPPV in the three groups respectively was 9, 21, and 12 patients. In the young group, 47 patients recurred 1 year after CRM treatment, and 28 patients (59.57%) suffered one episode of recurrence; six patients (12.76%) suffered two episodes of recurrence; and 13 patients (27.67%) suffered more than three episodes. In the middle-aged group, 117 patients recurred 1 year after CRM treatment, and 61 patients (52.13%) suffered one episode of recurrence; 17 patients (14.52%) suffered two episodes of recurrence; and 39 patients (33.35%) suffered more than three episodes. In the older group, 91 patients recurred 1 year after CRM treatment, and 53 patients (58.24%) suffered one episode of recurrence; 12 patients (13.18%) suffered two episodes of recurrence; and 26 patients (28.58%) suffered more than three episodes. The recurrence rate of BPPV 1 year after CRM treatment was 22.79% in young patients, 23.92% in middle-aged patients, and 28.57% in older patients, showing no statistically significant difference (*p* = 0.195). The prevalence of Ménière's disease, sudden deafness, hypertension, hyperlipemia, coronary heart disease, diabetes, arterial plaque, and cerebral infarction in young group was significantly lower than middle-aged and older aged group.

**Table 1:** Baseline characteristics of the three groups (*N* = 1,012).

**Variables**	**18–45 years (*****n*** **=** **208)**		**45–60 years (*****n*** **=** **489)**		**Over 60 years (*****n*** **=** **315)**		***p*-value**
	**Total (*N)***	**Column (*%*)**	**Total (*N*)**	**Column (*%*)**	**Total (*N*)**	**Column (*%*)**	
Age, years (mean ± SD)	37.10 ± 6.47		53.63 ± 4.18		67.70 ± 5.88		–
Disease duration (mean ± SD)	23.93 ± 46.21		40.38 ± 20.78		30.90 ± 57.66		0.158
Male	80	38.64	146	29.85	106	33.65	0.074
1-year recurrence	47	22.79	117	23.92	91	28.89	0.195
MD	1	0.04	10	2.04	13	4.12	0.024
Sudden deafness	0	0.00	4	2.22	7	0.08	0.045
Migraine	9	0.43	15	3.06	5	1.58	0.187
Hypertension	20	9.61	130	26.58	126	40.00	<0.001
Hyperlipemia	20	9.61	135	27.60	111	35.23	<0.001
Diabetes	11	5.28	36	7.36	40	12.69	0.005
CHD	0	0.00	22	4.49	35	11.11	<0.001
Arterial plaque	8	3.84	109	22.29	141	44.76	<0.001
CI	3	1.44	10	2.04	30	9.52	<0.001

*SD, standard deviation; MD, Ménière's disease; CHD, coronary heart disease; CI, cerebral infarction*.

### Recurrence Risk Factor Analysis

There was no statistically significant difference of recurrence rate in the three groups. We performed both univariate and multivariable analyses to identify the possible recurrence risk factors for BPPV in all of the patients. The results were shown in Table 2. We chose gender, sudden deafness, Ménière's disease, migraine, and cerebrovascular risk factors as recurrence-related variables. It showed that BPPV recurrence was associated with gender, Ménière's disease, migraine, sudden deafness, hyperlipemia, and hypertension (Table 2). Multivariable logistic regression revealed that Ménière's disease (odds ratio = 6.009, 95% confidence interval: 2.489–14.507, *p* < 0.001), hypertension (odds ratio = 1.510, 95% confidence interval: 1.095–2.084, *p* = 0.012), migraine (odds ratio = 2.534, 95% confidence interval: 1.164–5.516, *p* = 0.019), and hyperlipemia (odds ratio = 1.419, 95% confidence interval: 1.024–1.968, *p* = 0.036) may be risk factors for the recurrence of BPPV (Table 3).

**Table 2:** Univariate analysis of BPPV recurrence-related risk factors.

**Variables**	**Recurrence**		**Odds ratio**	**95% CI**	***p-*value**
	**Yes**	**No**			
	***N* (%)**	***N* (%)**			
Gender			0.679	0.495–0.931	0.016
Male	68 (26.67)	264 (34.87)			
Female	187 (73.33)	493 (65.13)			
Ménière's disease			6.268	2.649–14.828	<0.001
Yes	16 (6.27)	8 (1.05)			
No	239 (93.73)	749 (98.95)			
Migraine			2.874	1.367–6.039	0.005
Yes	14 (5.49)	15 (1.98)			
No	241 (94.51)	742 (98.02)			
Sudden deafness			3.619	1.095–11.962	0.035
Yes	6 (2.35)	5 (0.66)			
No	249 (97.65)	752 (99.34)			
Hypertension			1.631	1.201–2.215	0.002
Yes	89 (34.90)	187 (24.70)			
No	166 (65.10)	570 (75.30)			
Hyperlipemia			1.549	1.136–2.112	0.006
Yes	84 (32.94)	182 (24.04)			
No	171 (67.06)	575 (75.96)			
Diabetes			1.143	0.698–1.871	0.596
Yes	24 (9.41)	63 (8.32)			
No	231 (90.59)	694 (91.68)			
CHD			1.167	0.643–2.119	0.611
Yes	16 (6.27)	41 (5.41)			
No	239 (93.73)	716 (94.59)			
Arterial plaque			0.728	0.531–0.997	0.142
Yes	77 (30.19)	181 (23.91)			
No	178 (69.81)	575 (76.09)			
CI			1.118	0.543–2.302	0.762
Yes	10 (3.92)	33 (4.35)			
No	245 (96.08)	724 (95.65)			

*CHD, coronary heart disease; CI, cerebral infarction*.

**Table 3:** Multivariate analysis of BPPV patients during 1-year follow-up.

**Variables**	**Odds ratio**	**95% Confidence interval**	***p*-value**
Gender	0.714	0.517–0.986	0.041
Ménière's diseases	6.009	2.489–14.507	<0.001
Sudden deafness	1.858	0.511–6.760	0.347
Hypertension	1.510	1.095–2.084	0.012
Migraine	2.534	1.164–5.516	0.019
Hyperlipemia	1.419	1.024–1.968	0.036

## Discussion

This study shows that BPPV patients with Ménière's disease have 6.009-fold higher risk of recurrence compared to those without Ménière's disease. BPPV patients with hypertension have a 1.510-fold higher risk of recurrence compared to those without hypertension. The analysis also reveals that BPPV patients with migraine have a 2.534-fold higher risk of recurrence compared to those without migraine. Previous studies found that comorbidity with hypertension increased the risk of BPPV recurrence (20). Another study showed that the occurrence of hypertension and diabetes in BPPV patients influenced the prognosis of the residual symptoms (21). A recent meta-analysis reported that Ménière's disease was a risk factor for the recurrence of BPPV after otolith reduction (22). This is in agreement with the findings of this study. The prevalence of vertigo increases with age, being higher in individuals over 60 years old (23). The function of semicircular canals and otoconia gradually decreases due to age-related demineralization. This may cause blood vessel and circulation abnormalities leading to obstruction of inner ear circulation. Vascular stress of the anterior vestibular artery may reduce blood flow to the labyrinth and hence cause serious damage to the macula and otoconia detachment (24). Ballester reported that vertigo in BPPV patients with Ménière's disease required longer time to recover (25). This phenomenon may also be influenced by the balance disorder between endolymphatic absorption and utricle (26). This study shows that 10.3% of BPPV occurred as a secondary effect of impaired inner ear function, 50.7% due to sudden deafness, and 28.9% due to Ménière's disease (13).

Studies have reported that cerebrovascular disease is a risk factor for the recurrence of BPPV. In the present study, analysis of cerebrovascular disease risk factors in BPPV patients reveals that patients with hyperlipemia and hypertension may have a higher recurrence rate. A study found that the recurrence rate increased with the number of comorbidities of BPPV (27). Multiple systemic diseases can cause the labyrinth disorder leading to more frequent otolith detachment, which prolongs the recovery (28). Hyperinsulinism may disrupt inner ear hemostasis and alter the ionic and metabolic characteristics of the stria vascularis (7). On the other hand, hyperglycemia increases vascular resistance by inhibiting nitric oxide-related vasodilation. Therefore, a combination of hypertension and diabetes may lead to tissue hypoxia and cochleovestibular degeneration (5). Arterial plaque indicates early atherosclerosis. This may trigger intravascular thrombosis and cause hypoperfusion of the inner circulation (6). Previously, it was reported that residual symptoms in BPPV patients suffering from hypertension, diabetes, heart disease, and ischemic encephalopathy did not heal without treatment. Frequent recurrence episodes of BPPV may affect patients' quality of life. Therefore, early recognition and prompt management of BPPV are important in resolving further discomforts.

This study shows that the 1 year recurrence rate in young patients is 22.79%. There may be some unrecognized symptoms in young adults. Previous research has not largely focused on exploring the risk factors in a young population. Here, we divided the patients into young, middle-aged, and older patients and compared their features. Although the recurrence rate among the three groups was not statistically different, the older patients with a combination of cerebrovascular comorbidities need more attention.

Our study indicates that the 1-year recurrence rate in older BPPV patients is 28.89%. This suggests that the incidence rate of residual dizziness is 61%, which persists beyond 2 weeks even after successful treatment of BPPV with CRM. This may increase unsteadiness and risk of falls (11). The severest damage caused by BPPV is falls-related injuries and disability. Falls may lead to high morbidity and mortality in older patients, and the morbidity rate ranges from 13 to 38% (29). BPPV is the most common cause of vertigo disorders and is a well-recognized risk factor for falls. The proportion of aging population has been increasing in many countries. Therefore, prevention of falls in older people requires the development of more effective strategies. A timed Get-up-and-Go test which can be performed in <10 s has been recommended (30). Therefore, considering the benefits and harms caused by falls risk screening in older BPPV patients, it is necessary to implement early detection, diagnosis, and treatment. Otherwise, exercise and physical therapies can facilitate timely prevention and clinical intervention.

This study has the following limitations. First, our study reviewed recovery patients that may omit refractory cases, especially those with anterior semicircular canal BPPV. Second, some potential risk factors such as vestibular migraine, osteoporosis, or psychological factors were not available in this database. Due to incompleteness of patients' information, the occurrence of head trauma was not assessed in this study. In addition, we grouped BPPV patients into three groups according to age, which may cause comparison bias. We advocate for further studies to provide further detailed insight into the mechanisms of BPPV recurrence.

## Conclusion

In conclusion, we find that Ménière's disease, migraine, hyperlipemia, and hypertension exhibit a higher risk of recurrence compared to age-matched patients without these diseases. This retrospective study indicates the association of BPPV recurrence and cerebrovascular risk factors and provides some insight into the falls risk evaluation and early prevention of older patients with BPPV.

## Data Availability Statement

The datasets generated for this study are available on request to the corresponding author.

## Ethics Statement

Ethical review and approval was not required for the study on human participants in accordance with the local legislation and institutional requirements. The patients/participants provided their written informed consent to participate in this study.

## Author's Note

The abstract of this paper was presented at the XXX Bárány meeting in Uppsala as a poster presentation with interim findings.

## Author Contributions

CZ conceived the study and design, conducted the experiment, and wrote the manuscript. XZ provided the data analysis, preparation of manuscript, and revised this manuscript. YJ conceived the study and design and edited the manuscript. YW, MC, and YC conducted acquisition of subjects and interpretation of data.

### Conflict of Interest

The authors declare that the research was conducted in the absence of any commercial or financial relationships that could be construed as a potential conflict of interest.

## Supplementary Material

Supplementary Figure 1 The flow chart.
