# Supplementary material for: Clinical Characteristics and Risk Factors for the Recurrence of Benign Paroxysmal Positional Vertigo
Source: Front Neurol. 2019 Nov 13;10:1190. doi: 10.3389/fneur.2019.01190 (PMC6863975; doi:10.3389/fneur.2019.01190)
Supplement: Supplementary file 1 [file Table_1.DOCX]

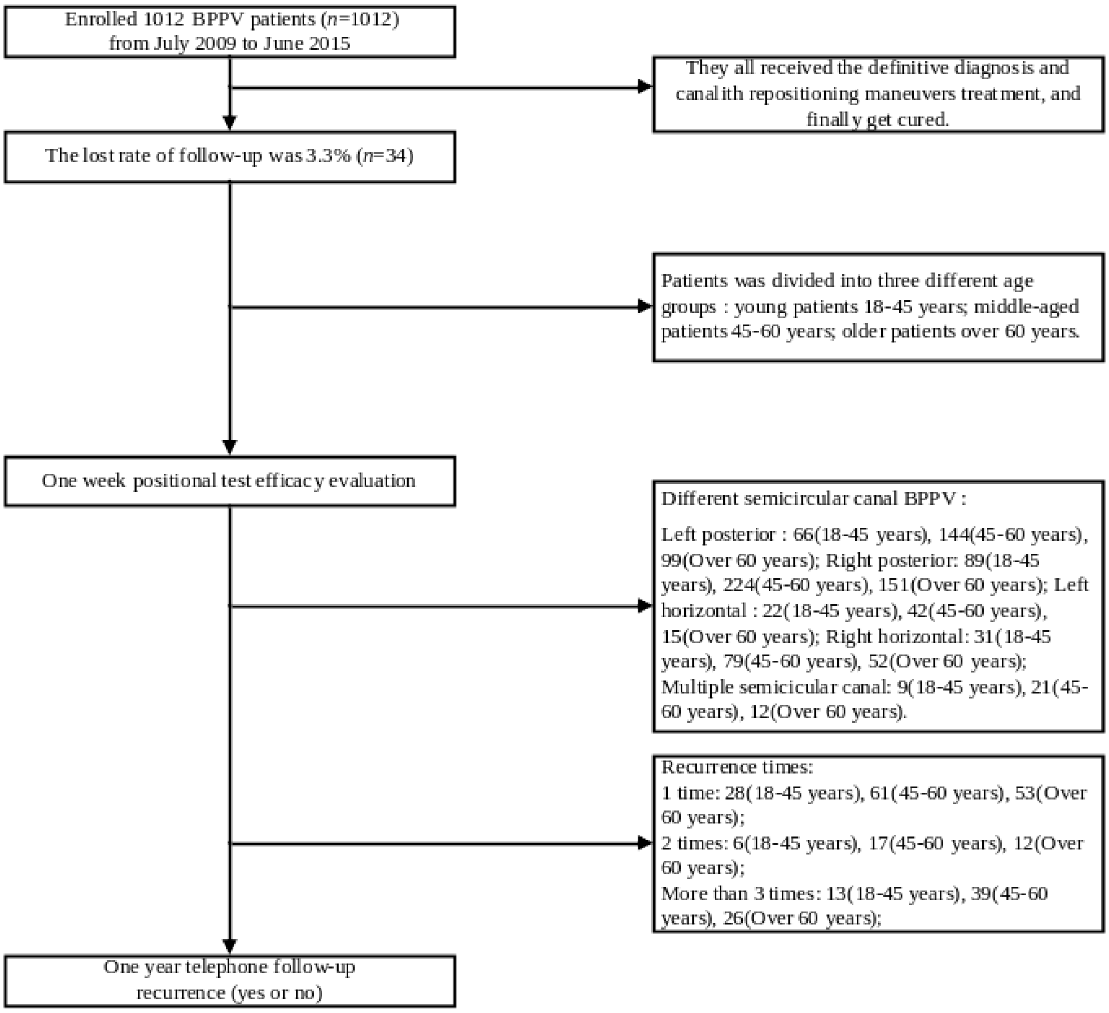
Enrolled 1012 BPPV patients (n=1012) from July 2009 to June 2015
They all received the definitive diagnosis and canalith repositioning maneuvers treatment, and finally get cured.
The lost rate of follow-up was 3.3% (n=34)
Patients was divided into three different age groups : young patients 18-45 years; middle-aged patients 45-60 years; older patients over 60 years.
One week positional test efficacy evaluation
Different semicircular canal BPPV :
Left posterior : 66(18-45 years), 144(45-60 years), 99(Over 60 years); Right posterior: 89(18-45 years), 224(45-60 years), 151(Over 60 years); Left horizontal : 22(18-45 years), 42(45-60 years), 15(Over 60 years); Right horizontal: 31(18-45 years), 79(45-60 years), 52(Over 60 years); Multiple semicicular canal: 9(18-45 years), 21(45-60 years), 12(Over 60 years).
Recurrence times:
1 time: 28(18-45 years), 61(45-60 years), 53(Over 60 years);
2 times: 6(18-45 years), 17(45-60 years), 12(Over 60 years);
More than 3 times: 13(18-45 years), 39(45-60 years), 26(Over 60 years);
One year telephone follow-up recurrence (yes or no)
